# Corneal Endothelial Graft Failure After Endoscopic Cyclophotocoagulation: A Case Report

**DOI:** 10.7759/cureus.72407

**Published:** 2024-10-26

**Authors:** Katsuhiko Maruyama, Masaki Tanito, Takefumi Yamaguchi, Jun Shimazaki

**Affiliations:** 1 Ophthalmology, Yashio Maruyama Eye Clinic, Saitama, JPN; 2 Ophthalmology, Shimane University Faculty of Medicine, Izumo, JPN; 3 Ophthalmology, Tokyo Dental College Ichikawa General Hospital, Ichikawa, JPN; 4 Ophthalmology, Akasaka Shimazaki Eye Clinic, Tokyo, JPN

**Keywords:** bullous keratopathy, complication, corneal edema, corneal transplantation, descemet's stripping automated endothelial keratoplasty, endoscopic cyclophotocoagulation, glaucoma surgery, graft failure, intraocular pressure (iop)

## Abstract

We report a case of corneal endothelial graft failure that developed after endoscopic cyclophotocoagulation (ECP) for elevated intraocular pressure (IOP) following Descemet's stripping automated endothelial keratoplasty (DSAEK). The patient was a 69-year-old Japanese woman with primary angle-closure glaucoma who had undergone phacoemulsification with intraocular lens implantation and goniosynechialysis for peripheral anterior synechiae (PAS), followed by trabeculectomy, repeat bleb revisions, Baerveldt glaucoma implant surgery, and Ahmed glaucoma valve implantation with tube insertion into the anterior chamber in the past two years. Subsequently, she developed bullous keratopathy, and the first DSAEK was performed in July 2020. Subsequently, iris adhesion and atrophy progressed around the tube, and PAS became severe, resulting in graft failure. A second DSAEK combined with pupilloplasty was performed in November 2021, after which the graft transparency was maintained. Since March 2022, IOP has increased despite treatment with maximum medication; therefore, ECP was performed to reduce IOP in September 2022. IOP decreased after ECP; however, the patient developed graft failure within a few months. A third DSAEK was performed in July 2023. In conclusion, for eyes with borderline corneal endothelial cell decompensation, the indications for ECP should be decided with caution.

## Introduction

Endoscopic cyclophotocoagulation (ECP) is a procedure that aims to reduce intraocular pressure (IOP) by directly irradiating the ciliary processes with a laser under endoscopic observation [1-5]. Compared with trans-scleral cyclophotocoagulation, which has a risk of overexposure because indirect laser irradiation is used, ECP has a lower incidence of complications such as phthisis and hypotony [6-8]. In addition, ECP that requires only a small incision in the limbus can be performed relatively easily, even in eyes with refractory glaucoma with scars in the conjunctiva and sclera caused by multiple surgeries.

However, compared to the low invasiveness indicated by the observed ocular findings, intraocular changes induced by ECP may be substantial. After ECP, aqueous humor production from the ciliary body decreases, which may change the composition of the aqueous humor and affect the function of corneal endothelial cells. We encountered a case of corneal endothelial graft failure after ECP following Descemet's stripping automated endothelial keratoplasty (DSAEK).

## Case presentation

The patient was a 69-year-old Japanese woman with primary angle-closure glaucoma who was already blind in her left eye. Due to a history of bronchial asthma and local eye allergy, the patient could only use prostaglandin FP receptor agonists and not any other IOP-lowering eye drops. The surgical history of the right eye is summarized as follows: she underwent phacoemulsification with intraocular lens implantation and goniosynechialysis for peripheral anterior synechiae (PAS) in November 2016, trabeculectomy in December 2016, bleb revision in February 2017, Baerveldt glaucoma implant (BGI) surgery in the temporal-inferior quadrant with tube insertion into the anterior chamber in April 2017, and Ahmed glaucoma valve (AGV) implantation in the temporal-superior quadrant with tube insertion into the anterior chamber in July 2017.

**Figure 1:**
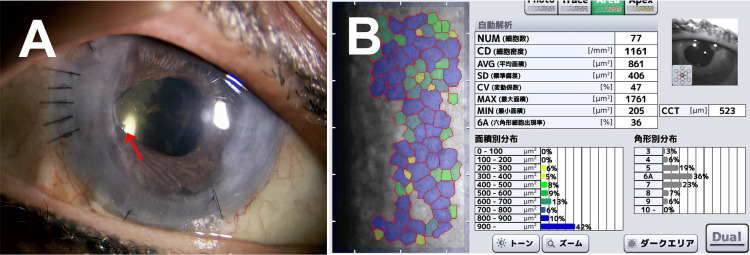
Slit lamp microscopy (A) and specular microscopy (B) findings after the second Descemet's stripping automated endothelial keratoplasty combined with pupilloplasty. (A) Graft transparency is maintained. The tube of the Baerveldt glaucoma implant was initially inserted into the anterior chamber from the temporal-inferior side. However, after pupilloplasty, the tip of the tube is visible behind the iris at 8 o'clock. (B) The corneal endothelial cell size is slightly irregular, but the cell density is 1,161, and the central corneal thickness is 523 micrometers. The red arrow indicates the tip of the Baerveldt glaucoma implant. NUM: number of cells, CD: cell density, AVG: average area, SD: standard deviation, CV: coefficient of variation, MAX: maximum area, MIN: minimum area, 6A: hexagonal cells, CCT: central corneal thickness.

Subsequently, the tip of the AGV tube inserted from the temporal-superior side came into contact with the corneal endothelium, and the AGV was removed in July 2018. As a result, the patient developed bullous keratopathy, and the first DSAEK was performed in July 2020. Then, iris adhesion and atrophy progressed around the BGI tube inserted into the anterior chamber from the temporal-inferior side, and PAS became severe, resulting in graft failure. Therefore, a second DSAEK combined with pupilloplasty was performed in November 2021. Subsequently, graft transparency was maintained (Figure 1A). The corneal endothelial cell size was slightly irregular but measurable by specular microscopy, and there was no corneal edema (Figure 1B). The best-corrected visual acuity (BCVA) was 20/40, and IOP was 13-15 mmHg without anti-glaucoma medication.

Since March 2022, IOP has increased to 20-25 mmHg despite treatment with latanoprost eye drops. At this time, BCVA remained unchanged at 20/40, and the corneal endothelial graft remained clear. In September 2022, ECP was performed to reduce the IOP. In this surgery, the laser power and duration were set at 200 mW and three seconds, respectively. Three-fourths of the circumference of the ciliary process was irradiated with a laser.

**Figure 2:**
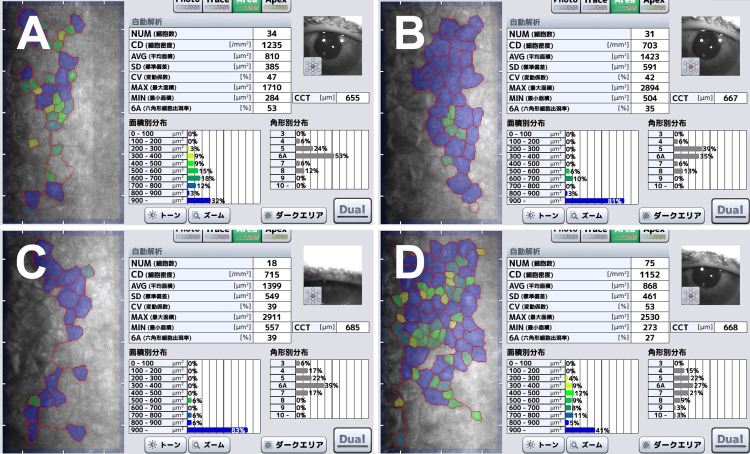
Specular microscopy at one (A), two (B), three (C), and four (D) months after endoscopic cyclophotocoagulation. The cell density is variable but measurable each month. The cornea remains thick. NUM: number of cells, CD: cell density, AVG: average area, SD: standard deviation, CV: coefficient of variation, MAX: maximum area, MIN: minimum area, 6A: hexagonal cells, CCT: central corneal thickness.

After ECP, the IOP decreased to 12-14 mmHg; however, corneal edema exacerbated immediately. Although corneal transparency was barely maintained and BCVA remained at 20/50 to 20/40, the thickened cornea did not improve at one month (Figure 2A), two months (Figure 2B), three months (Figure 2C), or four months (Figure 2D) after ECP.

**Figure 3:**
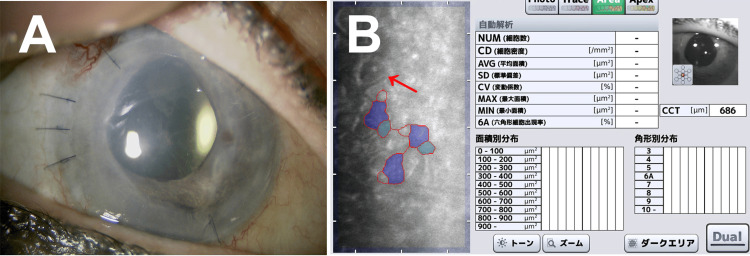
Slit lamp microscopy (A) and specular microscopy (B) findings five months after endoscopic cyclophotocoagulation. (A) The corneal endothelial graft is cloudy due to edema. (B) Corneal endothelial cells are enlarged, and the corneal thickness is thickened. Note that dark areas are observed inside some cells. The red arrow indicates the dark area inside the corneal endothelial cells. NUM: number of cells, CD: cell density, AVG: average area, SD: standard deviation, CV: coefficient of variation, MAX: maximum area, MIN: minimum area, 6A: hexagonal cells, CCT: central corneal thickness.

Five months after ECP, the corneal endothelial graft was cloudy due to edema, and the BCVA decreased to 20/100 (Figure 3A). Enlargement of the corneal endothelial cells and thickening of the cornea were observed using specular microscopy. In some cells, dark areas were observed inside the corneal endothelial cells (Figure 3B).

**Figure 4:**
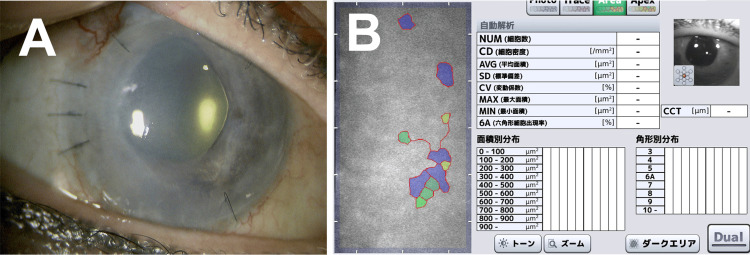
Slit lamp microscopy (A) and specular microscopy (B) findings nine months after endoscopic cyclophotocoagulation. (A) Corneal endothelial graft transparency is severely compromised. (B) None of the parameters are measured. NUM: number of cells, CD: cell density, AVG: average area, SD: standard deviation, CV: coefficient of variation, MAX: maximum area, MIN: minimum area, 6A: hexagonal cells, CCT: central corneal thickness.

Nine months after ECP, graft transparency was severely compromised, and BCVA decreased further to 20/200 (Figure 4A). All parameters of specular microscopy were not measurable (Figure 4B). A third DSAEK was performed in July 2023.

## Discussion

Herein, we report a case of corneal endothelial graft failure that developed after ECP for elevated IOP following DSAEK. This case presented risk factors of graft failure with multiple glaucoma surgeries, including prior implantations of glaucoma drainage devices [9]. Additionally, this eye had undergone multiple DSAEKs. Although it is unlikely that ECP independently caused graft failure, it is clear that ECP triggered graft failure since corneal edema was enhanced immediately after ECP was performed.

It is challenging to treat eyes with refractory glaucoma that have already undergone multiple surgeries. Surgeons often struggle with the choice of procedure. Against this background, it may be technically easier to apply ECP to eyes with scarring in the conjunctiva and sclera because only small incisions in the limbus are required.

However, compared with the low invasiveness indicated by the observed ocular findings, intraocular changes induced by the destruction of the ciliary body may be substantial. A previous study reported elevated concentrations of inflammatory cytokines in the aqueous humor in eyes with bullous keratopathy [10]. Furthermore, another report showed that changes in aqueous humor composition associated with iris atrophy predispose to the early development of graft failure [11]. In the present case, ECP was performed on an eye with severe PAS, iris injury including pupilloplasty, and a history of multiple DSAEKs. As a result, the suppression of aqueous humor production and disruption of the blood-aqueous barrier could lead to changes in aqueous humor composition and stagnation of aqueous humor circulation, which may have affected corneal endothelial cell viability and graft survival.

## Conclusions

We report a case of corneal endothelial graft failure after ECP following DSAEK. Although this case had risk factors for graft failure, including multiple glaucoma surgeries and DSAEKs, it was suggested that ECP was a trigger for graft failure because the corneal condition worsened immediately after ECP. For eyes with borderline corneal endothelial cell decompensation, the indication for ECP should be cautiously determined.

## References

[1] Uram M (1992). Ophthalmic laser microendoscope endophotocoagulation. Ophthalmology.

[2] Chen J, Cohn RA, Lin SC, Cortes AE, Alvarado JA (1997124). Endoscopic photocoagulation of the ciliary body for treatment of refractory glaucomas. Am J Ophthalmol.

[3] Murthy GJ, Murthy PR, Murthy KR, Kulkarni VV, Murthy KR (2009). A study of the efficacy of endoscopic cyclophotocoagulation for the treatment of refractory glaucomas. Indian J Ophthalmol.

[4] Kaplowitz K, Kuei A, Klenofsky B, Abazari A, Honkanen R (2015). The use of endoscopic cyclophotocoagulation for moderate to advanced glaucoma. Acta Ophthalmol.

[5] Tanito M, Manabe SI, Hamanaka T, Sato H, Mori K (2020). A case series of endoscopic cyclophotocoagulation with 532-nm laser in Japanese patients with refractory glaucoma. Eye (Lond).

[6] Ishida K (2013). Update on results and complications of cyclophotocoagulation. Curr Opin Ophthalmol.

[7] Anand N, Klug E, Nirappel A, Solá-Del Valle D (2020). A review of cyclodestructive procedures for the treatment of glaucoma. Semin Ophthalmol.

[8] Alasbali T (2023). Endoscopic cyclophotocoagulation for glaucoma compared to alternative procedures -a systematic review. Oman J Ophthalmol.

[9] Anshu A, Price MO, Price FW (2012). Descemet's stripping endothelial keratoplasty: long-term graft survival and risk factors for failure in eyes with preexisting glaucoma. Ophthalmology.

[10] Suzuki N, Yamaguchi T, Shibata S, Nagai T, Noma H, Tsubota K, Shimazaki J (2019). Cytokine levels in the aqueous humor are associated with corneal thickness in eyes with bullous keratopathy. Am J Ophthalmol.

[11] Yamaguchi T, Higa K, Yagi-Yaguchi Y (2020). Pathological processes in aqueous humor due to iris atrophy predispose to early corneal graft failure in humans and mice. Sci Adv.

